# Exploring the Molecular Tapestry: Organ-Specific Peptide and Protein Ultrafiltrates and Their Role in Therapeutics

**DOI:** 10.3390/ijms25052863

**Published:** 2024-03-01

**Authors:** Jakub Peter Slivka, Chris Bauer, Alexander Younsi, Michelle B. F. Wong, Mike K. S. Chan, Thomas Skutella

**Affiliations:** 1 Reviva, Plzenska 47, 252 19 Chrastany, Czech Republic; jakub.slivka@reviva.info; 2 MicroDiscovery, 10405 Berlin, Germany; 3 Department of Neurosurgery, Heidelberg University Hospital, 69120 Heidelberg, Germany; 4 Stellar Biomolecular Research GmbH, Klosterstrasse 205a, 67480 Edenkoben, Germany; 5 EW European Wellness International GmbH, Sommerhalde 21, 72184 Eutingen im Gäu, Germany; 6 Institute for Anatomy and Cell Biology, Medical Faculty, University of Heidelberg, Im Neuenheimer Feld 307, 69120 Heidelberg, Germany

**Keywords:** small proteins, ultrafiltration, proteomics, organ specificity

## Abstract

This study aims to characterize the proteome composition of organ-derived protein extracts from rabbits. Protein isolation was performed using soft homogenization and size exclusion via ultrafiltration. The proteome analysis of the ultrafiltrates was conducted using gel electrophoresis, and the mass spectrometry data were subjected to gene ontology analysis. Proteomic profiling revealed comprehensive protein profiles associated with RNA regulation, fatty acid binding, inflammatory response, oxidative stress, and metabolism. Additionally, our results demonstrate the presence of abundant small proteins, as observed in the mass spectrometry datasets. Small proteins and peptides are crucial in transcription modulation and various biological processes. The protein networks identified in the ultrafiltrates have the potential to enhance and complement biological therapeutic interventions. Data are available via ProteomeXchange with identifier PXD050039.

## 1. Introduction

The analysis of organ-specific peptide and protein (OSP) ultrafiltrates involves an extensive exploration of the complete protein composition in filtered organ or tissue solutions. This method facilitates identifying and characterizing proteins, peptides, and protein fragments within ultrafiltrates, offering several advantages in discovering clinically relevant molecules. Examining the protein makeup enables the identification of therapeutic targets and signaling molecules crucial in biological processes and disease treatment. Specific proteins, peptides, or protein fragments in ultrafiltrates provide insights into underlying molecular mechanisms associated with physiological and pathophysiological states. Techniques like gel electrophoresis, liquid chromatography, mass spectrometry, and bioinformatics enable protein separation, quantification, and sequencing, along with detecting post-translational modifications and protein–protein interactions.

Upon identification of proteins of interest through proteomic analysis, further investigations explore their functional roles, interactions, and potential clinical applications. Small proteins (SPs) and peptides, with potential therapeutic implications, are of medical interest within ultrafiltrates. SPs, genome-encoded protein products with up to 150 amino acids, act as intracellular modifiers, influencing cellular physiology and adaptability [1]. Due to their size and molecular weight, SPs are mass-produced and distributed for use in treatments, known as therapeutic proteins or peptides [2].

Decades of development have successfully utilized therapeutic SPs, also termed nano organo-specific peptides (NOP), for various indications, given their antimicrobial, antioxidative, and immunomodulatory properties [3]. For example, thymus peptides restore T-cell functions, while peptides from placenta and protein hydrolysates exhibit high antioxidant activity [4,5]. SP therapy utilizing organ-specific extracts targets diseased or aging tissue, emphasizing the need to explore and characterize potential organ-specific proteins for clinical use.

Advancements in bioinformatics and synthetic techniques open new possibilities for developing protein therapies, ushering in a new era for therapeutic SPs. These SPs are used in clinical trials for conditions like cancer, diabetes, autoimmune responses, neurodegenerative, and cardiovascular diseases. For example, RNA-binding proteins (RBPs) have been known to play a key role in regulating a broad spectrum of cellular processes; however, recent studies on small RBPs have shed light on the therapeutic potential of RBPs for various indications. These findings suggest the possible involvement and mechanisms of RBPs in anti-aging [6,7] and anti-cellular senescence [8]. Similarly, spliceosome and small protein-regulated lipid metabolism also exhibit positive regulation in aging and cellular senescence [9,10]. These show that SPs could help aging-related diseases and promote longevity in humans.

Understanding the function and networks of SPs may lead to their use as supplements or therapeutics, supporting physiological and pathological conditions. Researchers and pharmaceutical companies actively pursue the search and development of bioactive SPs.

Therapeutic SPs can be categorized based on pharmacological activities, such as replacing deficient proteins, augmenting existing pathways, providing novel functions, or interfering with specific molecules or organisms. Our study aimed to gain a deeper understanding of the proteome, focusing on the possible therapeutic effects of SPs. As a result, these therapeutic SPs have been used widely in clinical trials for cancer, diabetes, autoimmune response, antimicrobial, neurodegenerative, and cardiovascular diseases [11].

Monoclonal antibodies and cytokines are examples of some of the macromolecular therapeutic proteins [12]. The success of the commercialization of Liraglutide and Semaglutide for the treatment of type 2 diabetes and obesity, which exceeded USD 7.6 billion in revenues in 2020, is one of the factors driving the development of these therapeutic proteins/peptides to enter clinical trials [13]. We isolated OSPs from selected organs in rabbits, identifying their contents through Liquid Chromatography–Mass Spectrometry/Mass Spectrometry (LC-MS/MS). Employing a general bottom-up proteomics approach, our objective is to gain functional insights into these organ/tissue extracts and comprehend their potential for use in therapeutic applications. The proteomic analysis of OSPs represents a focused inquiry into the complex protein landscape within filtered organ or tissue solutions. The rationale behind choosing OSPs as the subject of the study lies in the unique insights they offer into the molecular intricacies governing various physiological and pathophysiological states. We sought to address a central thesis: to unravel the comprehensive proteomic profile of OSPs and harness this knowledge to identify therapeutic targets, signaling molecules, and organ-specific proteins with potential clinical applications.

By isolating and analyzing OSPs, the study aims to characterize the entire protein complement and unravel the potential functional roles and interactions of identified proteins. A comprehensive proteomic approach involving techniques was intended to delineate the intricate details of the OSP proteome and explore the therapeutic implications of SPs and peptides within ultrafiltrates. Recognizing SPs as genome-encoded protein products with unique roles in cellular physiology and adaptability, the study aims to shed light on their potential use as therapeutic proteins or peptides. The short length and low molecular weight of SPs make them amenable to mass production and distribution, opening up avenues for their utilization in treatments.

The potential for developing novel protein therapies, including proteins and small proteins, could be foreseen in advancing technology, particularly in bioinformatics and synthetic techniques. The study acknowledges the growing significance of these therapeutic SPs in various clinical trials for conditions ranging from cancer to neurodegenerative diseases. By understanding the function and networks of SPs, it is believed that these molecules could serve as essential regulators in cellular and intercellular physiological processes, prompting researchers and pharmaceutical companies to actively pursue their discovery and development.

## 2. Results

### 2.1. Characterization of OSP Ultrafiltrates from Rabbit Whole Tissue and Organs

**Figure 1:**
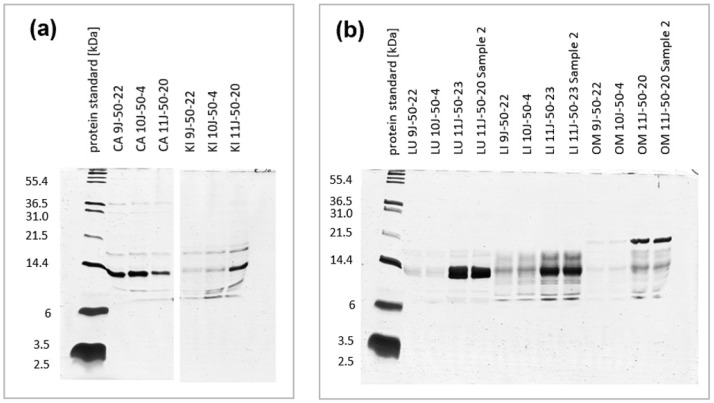

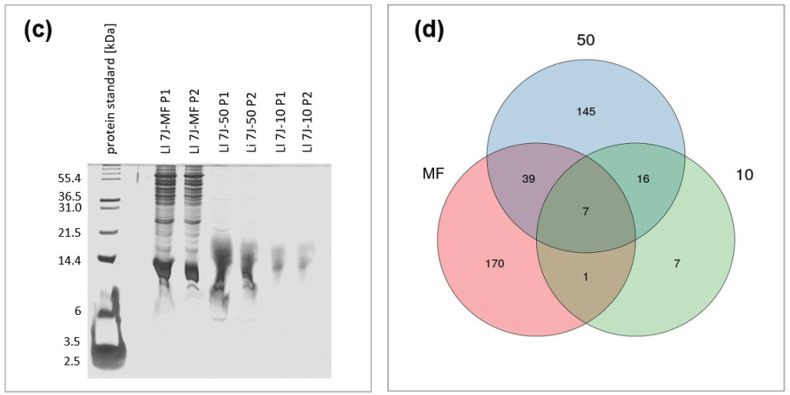
Tris-Tricine-PAGE of OSPs obtained from ultrafiltration: (**a**) intercostal muscle (IM) and kidney (KI), runs 9J, 10J, and 11J, two lanes were cut (represented with a gap in the gel picture) from the picture due to the samples not being a part of this experiment; (**b**) lung (LU), liver (LI), and organ mixture (OM), runs 9J, 10J, and 11J; (**c**) Tris-Tricine-PAGE of liver tissue extracts obtained by microfiltration (MF: MWCO 0.2 um) and different ultrafiltration (UF1: MWCO 50 kDa, UF2: MWCO 10 kDa); (**d**) number of proteins/peptides selected by the different selection procedures (right).

In this study, we aimed to optimize the extraction of different OSPs. A specific extraction protocol was employed for postnatal rabbit organs/tissues. In brief, the organs/tissues were homogenized and lysed using mechanical disruption, followed by pre-filtration and ultrafiltration steps. Three approaches were utilized to gain insights into the protein content and characteristics of the various ultrafiltrates: the Bradford assay, protein gels, and mass spectrometry. Our findings revealed the presence of small protein/peptide fractions in the ultrafiltration, with sizes ranging from approximately 5 to 50 kDa. The Bradford method measured 0.1–5 µg/µL of small proteins and peptides in the ultrafiltrates. Additionally, Tris-Tricine-PAGE gels, known for their superior ability to separate small proteins and peptides compared to SDS-PAGE gels, were employed to validate the size distribution of proteins in the different ultrafiltrates. The results obtained from the Tris-Tricine-PAGE gels exhibited a significant enrichment of bands and clear smears within the 10 kDa to 20 kDa fractions (Figure 1a–c).

A shift in the sample size (Figure 1c) and numbers (Figure 1d) of proteins was observed. In the groups from the microfiltration with 0.2 um, we observed dense bands ranging from 10 kDa to 55 kDa and higher. In the 50 kDa samples, the major protein bands ranged between 6 and 20 kDa, with a dense band pattern around 14 kDa. On the other hand, in the 10 kDa samples, a smear of lower intensity in comparison to the 50 kDa samples was observed between 10 and 15 kDa. Similarly, according to initially performed mass spectrometry runs, the number of detected proteins in the corresponding fractions decreased from 170 (MF) to 145 (UF1) to 7 (UF2) detectable proteins (Figure 1d). Due to our focus on the enrichment of small proteins, we continued from this point to the analysis with the 50 kDa samples (UF1). 

**Figure 2:**
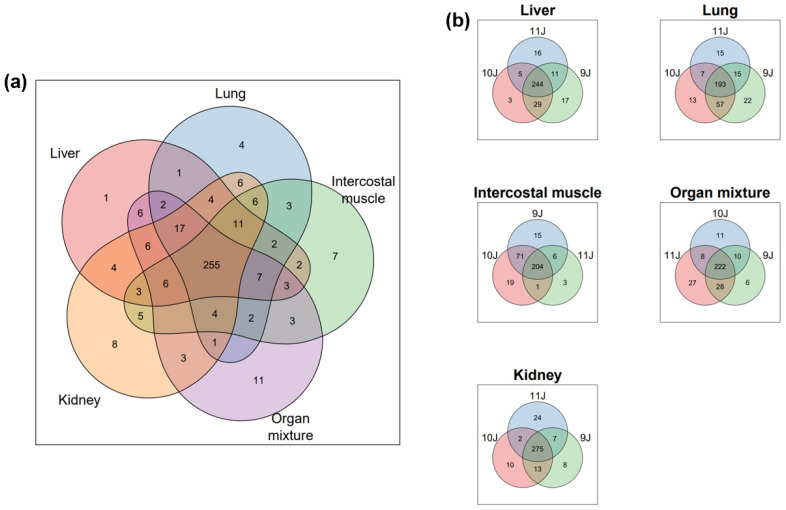
Venn diagram comparing the identified proteins (**a**) in at least one tissue of the four experiments with a total number of proteins measured is 393; (**b**) of the same tissues (liver, lung, intercostal muscle, organ Mixture, and kidney) between the experiments.

To define protein/SPs signatures in each organ, we performed a comprehensive functional analysis of the proteomics datasets to investigate functional protein networks. To compare the three experiments, we generated a Venn diagram for overlapping proteins in at least one sample of the experiment, as illustrated in Figure 2. We found a total of 393 proteins measured in at least one experiment, indicating a relatively high overlap between the different datasets. Among these proteins, approximately 255 (64.89%) were identified in at least one tissue across all datasets. Interestingly, we observed a substantial proportion of uniquely annotated categories for each OSP ultrafiltrate, indicating distinct organ-specific protein profiles. These annotated categories reflected their respective organ sources, as illustrated in Figure 2b.

**Figure 3:**
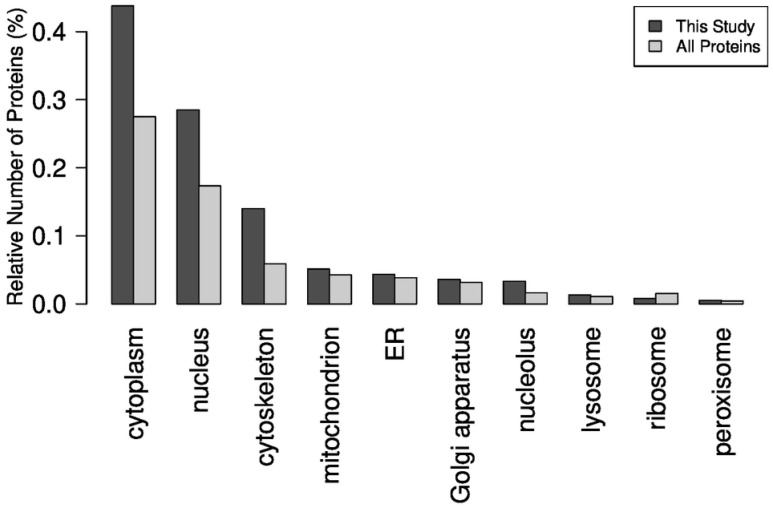
Cellular compartments of the fractioned proteins of OSPs.

As became evident, the highest proportion of proteins is related to the cellular compartments of the cytoplasm, nucleus, and cytoskeleton, followed by mitochondria (Figure 3). Consequently, we aimed to provide a comprehensive overview of the enriched proteome of protein and peptides found in OSPs of four different rabbit tissue/organs (intercostal muscle, lung, liver, kidney) and in rabbit organ mixtures consisting of liver, pancreas, placenta, stomach, intestinal mucosa, kidney, and eye (OM), to obtain insights into the Gene Ontological (GO) molecular processes involved in OSPs.

### 2.2. OSPs Revealed GO Enrichment in RNA Processing, Fatty Acid Binding, Oxidoreductase Activity, Cyclosporin A Binding and Significant KEGG Pathways

**Figure 4:**
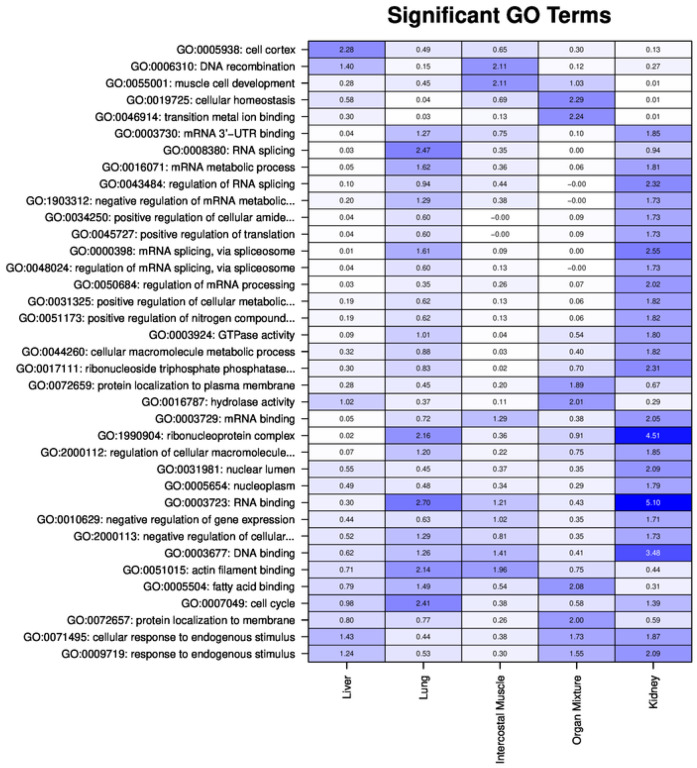
Matrix with −log_10_ *p*-values for significantly identified GO terms (*p*-value < 10^−5^) for the different tissues of the new dataset. Columns show the different organs/tissues; rows show the GO terms. Columns are ordered by organ/tissue. For the GO analysis, we used differentially regulated proteins (absolute fold change > 2) in any of the tissues.

Gene Ontology (GO) analysis of the OSPs showed a distinct general enrichment in proteins significantly and particularly associated with RNA processing (“RNA binding”, “spliceosome complex”), DNA processing, and “fatty acid binding” (Figure 4). 

“RNA-binding” showed the highest enrichment in OSP from the kidney, followed by lung and IM. The results indicate the presence of highly involved proteins in RNA binding, activation, or modification. Subsequently, GO terms related to the spliceosomal regulation splicing of RNA were enriched in the lung OSPs. The GO term refers to the formation of ribonucleoprotein (spliceosome) from small nuclear RNAs (snRNAs) and small nuclear ribonucleoproteins (snRNPs). According to Figure 4, “Fatty acid binding” is most enriched in the OSPs from OM, followed by lung and liver. 

**Figure 5:**
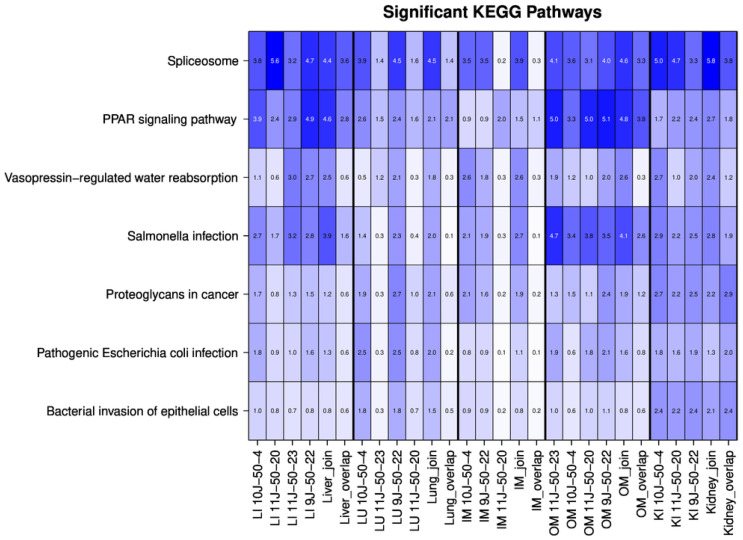
Matrix with −log_10_ *p*-values for significantly identified KEGG pathways (pFDR < 10^−3^) for the different samples from the new dataset. Columns show the different organs/tissues; rows show the pathways. We used all pathways with a pFDR value < 0.001 in any of the tissues.

The KEGG analysis revealed strong enrichment in the PPAR signaling pathway and spliceosome for OSPs, as shown in Figure 5. The “PPAR signaling pathway” was highly enriched in all OSP tissues except IM, where the enrichment was not as high. The spliceosome in KEGG was strongly related to the ultrafiltrates of the liver, OM, and kidney (Figure 5). Other significant KEGG pathways in all samples were related to immune response and activation/modulation. These were significant in kidneys and some in OM samples.

### 2.3. Different Organs/Tissues of OSPs Displayed Various Functional Biological or GO Terms Using the Integration of Proteomic (Mass Spectrometry) and the Bioinformatic Technique 

**Figure 6:**
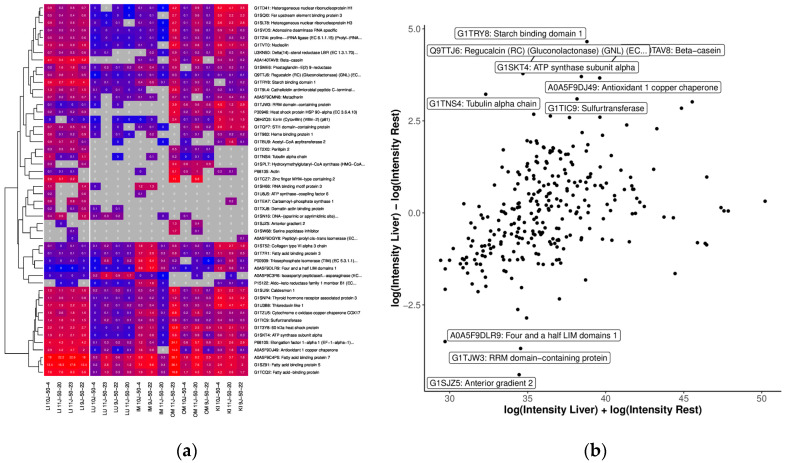
Comparison of OSP abundance with (**a**) heatmap of the top 50 proteins with the highest differences between the tissue of the liver and the other tissues. We only considered proteins measured in at least 50% of the liver samples and 33% of the other samples. The grey color indicates missing values (protein was not measured). Clustering and coloring are based on the log-transformed LFQ intensities. We annotated the LFQ intensities (in millions) without logarithmic transformation in the cells for better interpretability. (**b**) MA plot of quantifications (LFQ intensities after logarithmic transform) of tissue: liver. The top 10 proteins (with the highest difference) are annotated.

The liver OSPs showed significant overall GO terms related to “platelet activation”, “regulation of body fluid levels”, and “lipid droplet” (Supplementary Table S2). We also analyzed each sample in the OSPs to compare their abundance of organ specific proteins based on protein IDs using LFQ intensities after logarithmic transformation to generate the MA plot and heatmap for quantification, as shown in Figure 6. In total, 330 of the proteins found in the liver were analyzed. Of these, 94 proteins were <20 kDa and 235 were <50 kDa, respectively. Proteins more expressed in liver than other tissue included Starch binding domain 1, Regucalcin, Sulfurtransferase, Antioxidant 1 copper chaperone and Fatty acid binding protein 5 and 7 (Figure 6a,b).

In the OSPs lung, we identified GO terms “innate immune response”, “regulation of wound healing”, “defense response”, and “regulation of response to wounding” for molecular function (Supplementary Table S2). The lung OSP contained proteins associated with endothelial and alveolar epithelial cells at about 28.2% (92 out of 325 proteins found), according to the HPA database. Of 325 proteins, 90 were <20 kDa, and 230 were <50 kDa. The most highly upregulated protein in the lung was found to be Isopropyl peptidase/L-asparinase. 

**Figure 7:**
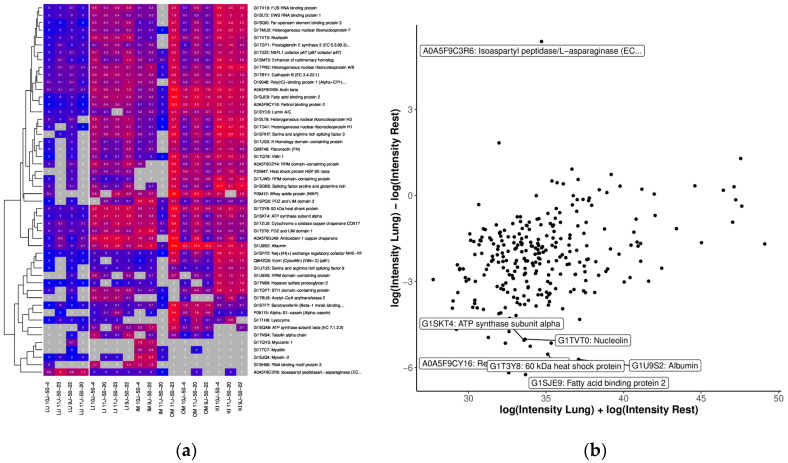
Comparison of OSP abundance with (**a**) heatmap of the top 50 proteins with the highest differences between tissue of the lung and the other tissues. We only considered proteins measured in at least 50% of the lung samples and 33% of the other samples. The grey color indicates missing values (protein was not measured). Clustering and coloring are based on the log-transformed LFQ intensities. For better interpretability, we annotated the LFQ intensities (in millions) without logarithmic transformation in the cells; (**b**) MA plot of quantifications (LFQ intensities after logarithmic transform) of tissue from the lung. The top 10 proteins (with the highest difference) are annotated.

**Figure 8:**
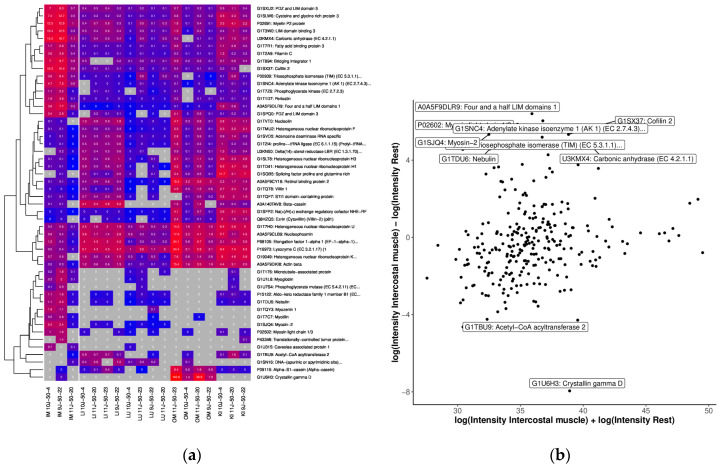
Comparison of OSP abundance with (**a**) heatmap of the top 50 proteins with the highest differences between tissue of IM and the other tissues. We only considered proteins measured in at least 50% of the IM and 33% of the other samples. The grey color indicates missing values (protein was not measured). Clustering and coloring are based on the log-transformed LFQ intensities. We annotated the LFQ intensities (in millions) without logarithmic transformation in the cells for better interpretability. (**b**) MA plot of quantifications for intercostal muscle (LFQ intensities after logarithmic transform) of tissue from intercostal muscle. The top 10 proteins (with the highest difference) are annotated.

In the category biological process, the GO terms “response to stress”, “calcium-dependent protein binding”, and “regulation of blood vessel endothelial cell migration” (Supplementary Table S2) revealed the largest unique alterations in the intercostal muscle OSP. Many skeletal muscle-related proteins were found in the proteome of rabbit intercostal muscle. HPA reported that 66, or approximately 20.5%, of the 319 different proteins and peptides that were found in intercostal muscle tissue were associated with the term “skeletal muscle” or “bone marrow”; 91 proteins were under 20 kDa, and 141 were under 50 kDa. In this intercostal muscle OSP, a strong enrichment of skeletal muscle-associated proteins was observed (Figure 7). Highly expressed proteins in the intercostal muscle OSPs included Myoglobin, Myotilin, Periostin, Bridging integrator 1, Cofilin 2, Adenylate kinase isoenzyme 1 and Myotropin (Figure 8). These protein networks were not detected in any other OSP in this proteomics study (Supplementary Table S1).

**Figure 9:**
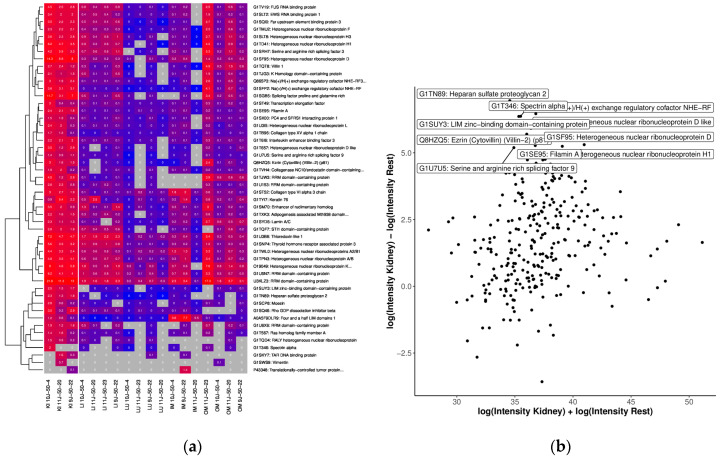
Comparison of OSP abundance with (**a**) heatmap of the top 50 proteins with the highest differences between tissue from kidney and the other tissues. We only considered proteins measured in at least 50% of the kidney samples and 33% of the other samples. The grey color indicates missing values (protein was not measured). Clustering and coloring are based on the log-transformed LFQ intensities. We annotated the LFQ intensities (in millions) without logarithmic transformation in the cells for better interpretability. (**b**) MA plot of quantifications (LFQ intensities after logarithmic transform) of tissue from the kidney. The top 10 proteins (with the highest difference) are annotated.

Significant GO terms for kidney OSP were “anatomical structure formation involved in morphogenesis”, “cellular component biogenesis”, “embryo development”, and “positive regulation of viral process”. Another significant term related significantly more to the kidney than other tissues was “angiogenesis” (Supplementary Table S2). According to kidney protein analysis, a total of 339 proteins were identified. Of these, 66 proteins were <20 kDa and 168 < 50 kDa. The kidney OSP also constituted a set of organ-specific enriched proteins (Figure 9a). From the 339 proteins that were identified, 115 were found to be associated with kidneys (Supplementary Table S2). Proteins found only in kidney OSP samples included Heparan sulfate proteoglycan 2, Heterogeneous nuclear ribonucleoprotein D, Ezrin, Spectrin alpha, and Filamin A (Figure 9b).

**Figure 10:**
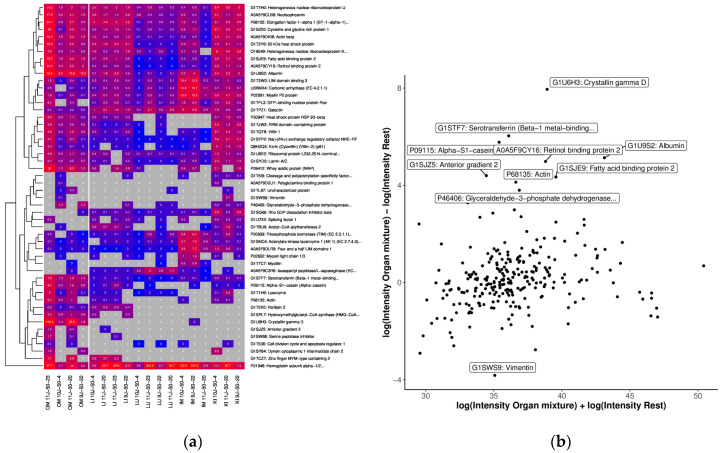
Comparison of OSP abundance with (**a**) heatmap of the top 50 proteins with the highest differences between tissue from OM and the other tissues. We only considered proteins measured in at least 50% of the OM samples and 33% of the others. The grey color indicates missing values (protein was not measured). Clustering and coloring are based on the log-transformed LFQ intensities. We annotated the LFQ intensities (in millions) without logarithmic transformation in the cells for better interpretability. (**b**) MA plot of quantifications (LFQ intensities after logarithmic transform) of tissue from OM. The top 10 proteins (with the highest difference) are annotated.

In the OM results, the most significant GO terms in the biological product were “protein dimerization activity”, “negative regulation of blood vessel endothelial cell migration”, and “C-acetyltransferase activity” (Supp. Table S2). There were 326 proteins identified, of which 154 unique proteins were related to some of the tissue from the OM OSP, i.e., out of 326 genes, 34 were related to the colon, 33 to the duodenum, 6 to the eye, 110 to kidney, 105 to the liver, 58 to the placenta, 32 to the small intestine, 72 to stomach and 62 to the pancreas. Of the 149 identified proteins, 93 were under 20 kDa, and 235 were under 50 kDa. We observed that retinol binding protein 2, fatty acid binding protein, Serotransferrin and alpha S1 casein were of high abundance in OM based on the MA plot (Figure 10b) and heatmap in Figure 10a. The proteins found only in OM samples were Galectin, Crystallin gamma N, Crystallin beta A1/B1/B3, DCC netrin receptor, small muscular protein, and Plectin. 

In addition, RBP2 Retinol-binding protein 2 (RBP2), fatty acid binding protein 2, 7.60 kDa heat shock protein, and Albumin were highly expressed in the OM OSP. Another protein we observed in the OM was lysozyme C, which was also present in the liver, lung, and kidney but was highly expressed mostly in the OM and kidney OSPs. 

The GO classifications of the unique proteins in each organ proteome suggest distinctive molecular signs in each OSP (Figure 6, Figure 7, Figure 8, Figure 9 and Figure 10). Our proteomics analysis shows enrichment in important functional proteins in rabbit organs, as confirmed by GO term analysis. 

### 2.4. Small Proteins in OSPs

**Figure 11:**
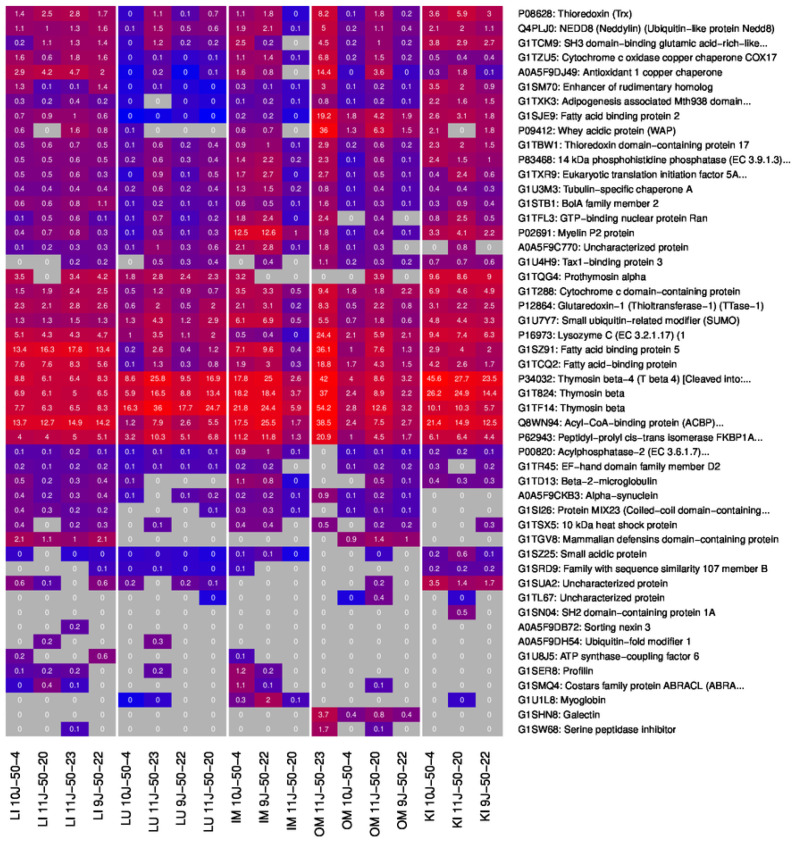
Heatmap of the top 50 small proteins (under 150aa) with their expression rates shown.

Apart from the tissue-specific proteins found in OSPs, many small proteins were identified across all the samples, although with different abundances and expression rates. In total, 69 unique SPs were detected in the samples. The percentage of small proteins from the totality of proteins found (396) was 17.68%; in the lungs, the ratio was the highest (20.9%), while in the kidneys, it was the lowest (19%). Figure 11 highlights the top 50 small proteins (under 150 aa) with the highest expressions, while the complete list is provided in Supplementary Table S3. Approximately 64% of the SPs were common in all samples. Only four SPs were uniquely found in a certain tissue (two in IM OSP and two in OM OSP). From 206 proteins found in all samples, 44, or 21%, were SPs. Therefore, the tissue-specific proteins were mostly not SPs but proteins with >150 amino acids. Although there was a significant overlap of small proteins across samples, certain small proteins exhibited markedly higher abundance in specific tissues, including Prothymosin alpha, Thymosin beta-4 in the kidney, Fatty Acid protein 5 in the liver, and Myelin P2 protein in IM, and Lysozyme C in the kidney.

The data from the table clearly indicate that the organ mix and intercostal muscle protein solutions contained the largest number of uniquely occurring proteins. Notably, Thioredoxin 2 was unique to the OSP from IM, whereas Galectin was only present in OM (see Table 1). Liver OSPs, on the other hand, had only one detectable unique protein or SP.

## 3. Discussion

This study is the first report on identifying and characterizing proteins and SPs in organ-specific ultrafiltrates from postnatal rabbits, which we abbreviated as OSPs, through the integration of proteomic approaches. 

Here, in the first step, we discuss enriched GO analysis and high-abundance proteins connected to the different OSPs and exhibit potential organ-specific activity due to their expression and/or abundance.

### 3.1. General Highly Abundant GO-Terms for OSPs

From our findings, the GO term “RNA binding” was preferentially enriched in the OSPs from all the organs except the lung and intercostal muscle. The RBPs included Heterogeneous nuclear ribonucleoproteins A1, K, A/B, and Park7. RBPs are known to be involved in each step of RNA metabolism and, therefore, are critical effectors involved in gene expression and regulation [14]. Several proteins are related to the GO term mentioned above. For instance, the eight proteins of hnRNPs from kidney analysis are RNA-binding proteins, and they form complexes with heterogeneous nuclear RNA (hnRNA) as the integral protein components [15]. These proteins are associated with pre-mRNAs in the nucleus and appear to influence pre-mRNA processing and other aspects of mRNA metabolism and transport [16]. Another interesting RNA binding protein from the kidney and other OSPs is the RNA binding protein fused in sarcoma/translocated in liposarcoma (FUS/TLS or FUS). FUS is a multifunctional DNA-/RNA-binding protein involved in various cellular functions, including transcription, protein translation, RNA splicing, and transport [17]. Besides, ELAV-like protein, a member of the ELAVL family of RNA-binding proteins from kidney tissue, contains several RNA recognition motifs and selectively binds AU-rich elements (AREs) found in the 3′ untranslated regions of mRNAs [18]. AREs signal the degradation of mRNAs to regulate gene expression; thus, by binding AREs, the ELAVL family of proteins plays a role in stabilizing ARE-containing mRNAs. “RNA binding” and “mRNA splicing via spliceosome” may play pivotal roles in metabolism or immune response. It has been shown how splicing controls the metabolic reprogramming of tumor cells as a different approach to treating cancer [19]. 

Concomitantly, a biological process, “mRNA splicing, via spliceosome”, which is involved in the removal of introns from a transcribed pre-mRNA and joining the exons to form a complete mRNA [20], was revealed to be enriched in the liver and kidney. The spliceosome is a macromolecular complex found within the nucleus of eukaryotic cells. A small nuclear ribonucleoprotein (snRNP) complex is first formed when several small nuclear RNA (snRNA) molecules bind together. It then combines with other snRNPs to form a large ribonucleoprotein complex called a spliceosome [21]. The spliceosome is known to remove introns from a transcribed pre-mRNA via splicing [22].

FABPs are a family of small, highly conserved, cytoplasmic proteins that bind long-chain fatty acids to facilitate movement across extracellular and intracellular membranes and other hydrophobic ligands [23]. These proteins are believed to transfer lipophilic substances such as eicosanoids and retinoids from the outside cell membrane to specific intracellular receptors, such as PPA receptors (PPAR), a group of nuclear receptor proteins [23]. By binding to PPAR-responsive regulatory elements as obligate heterodimers with retinoid X receptor (RXR), the PPARs control the expression of networks of genes involved in energy production, lipid metabolism, and inflammation [24,25]. The activation of PPAR-α reduces triglyceride levels and is involved in regulating energy homeostasis. The activation of PPAR-γ causes insulin sensitization and enhances glucose metabolism, whereas the activation of PPAR-β/δ enhances fatty acids metabolism [24]. One of the FBPs in our findings, Fatty Acid Binding Protein 5 (FABP5), expressed in macrophages, is suggested to sequester anti-inflammatory mediators from their target, creating a pro-inflammatory environment. In addition, FBPs’ protein levels have decreased with aging in the brain, which may be a factor in the decline in synaptic activity linked to aging [26], so more in-depth research for OSPs anti-aging via FBPs in the future is essential. 

The GO analysis revealed OSPs’ potential role in the immune system, inflammation, and oxidative stress. Various proteins related to these processes were identified, including Galectin, Glutaredoxin-1, Thioredoxin, Antioxidant 1 copper chaperone, COX 17, Thymosin beta, Thymosin beta 4, and Prothymosin alpha. These proteins exhibit diverse functions, such as anti-inflammatory effects, antimicrobial activities, and protection against bacterial infections. Additionally, the study explored oxidative stress-related proteins like metallothionein-2E, which are found in liver tissues and known for their antioxidant functions and heavy metal detoxification properties. The metabolism-related aspects included the enrichment of vitamin D binding and the involvement of the DBI diazepam binding inhibitor in lipid metabolism. Glycolytic processes in most OSPs featured key enzymes such as Phosphoglycerate kinase (PGK 1) and triose-phosphate isomerase (TPI or TIM). Furthermore, the analysis highlighted metabolism-associated proteins, such as retinol binding protein (RBP), RAD23, and Regucalcin. These proteins play roles in nucleotide excision repair, protein degradation, calcium homeostasis, and various physiological processes, including cell growth, apoptosis, hormone synthesis, and antioxidant defense. Regucalcin was noted for its potential protective effect against age-related diseases. It has also been studied for its anti-cancer properties, which could be how this protein is used [23].

### 3.2. Identification of Highly Expressed Small Proteins in the OSPs

Our investigation into OSPs has uncovered intriguing small-molecular-weight peptides such as Prothymosin alpha, Thymosin beta 4 (Tb4), Thymosin beta (TB), galectin and TAGLN, which have the potential to contribute to regenerative effects indirectly. Tb4 and thymosin beta (TB) play important roles in the immune system and tissue repair processes. While they share similarities in their names, they are distinct molecules with different functions. Tb4 is a naturally occurring peptide consisting of 43 amino acids. It is primarily produced by the thymus gland, a key organ in the immune system, but can also be found in other tissues. Tb4 has been found to have multiple roles in various physiological processes. Thymosin beta 4 (Tb4) and thymosin beta (TB) are important peptides in the immune system and tissue repair processes. While they share similarities in their names, they are distinct molecules with different functions. Studies have demonstrated that Tb4 promotes the survival of neurons and stimulates neurite outgrowth in cultured spinal cord neurons [24]. Furthermore, Tb4 exhibits angiogenic properties by enhancing endothelial cell (EC) migration, tubule formation, and angiogenesis [25]. It has also been observed that Tb4 facilitates the differentiation of epicardial progenitor cells into endothelial cells, serving as a source of vascular progenitors for coronary vasculogenesis and angiogenesis. Additionally, Tb4 plays a role in wound healing in the skin and cornea [26], and it has shown potential to improve heart function after myocardial infarction [27]. Recently, it has been shown that Tb4 and Prothymosin alpha can promote cardiac regeneration [28]. Prothymosin might have potential therapeutic applications in treating autoimmune diseases and cancer. Thymosin Beta (TB), also known as thymosin beta 15, is another peptide structurally related to Tb4 but has distinct functions. It consists of 15 amino acids and has been implicated in various cellular processes, including the cellular development of muscle cells. It also influences cell migration and has anti-apoptotic and neuroprotective properties [29,30]. It has shown potential neuroprotective effects in certain experimental models of neurodegenerative diseases. It has been found to promote neuronal survival and inhibit neurons’ apoptosis (programmed cell death). Tb4 has shown potential applicability in clinical trials in treating cardiovascular diseases, neurodegenerative disorders, corneal defects, and skin injuries (clinicaltrials.gov, accessed on 20 January 2024).

The small protein Galectin, most abundant in IM, plays a role in various biological processes, including cell adhesion, immune response regulation, and inflammation. Yang et al. (2023) also observed that Galactin-1 and Secernin function as axonal guidance molecules [31]. Galectins have been studied for their potential therapeutic applications against infection, inflammation, and immune disorders [32].

TAGLN and TAGLN 2, peptides derived from OSPs, are well-known for their function as actin-crosslinking/gelling proteins belonging to the calponin family. TAGLN is primarily localized in the cytoskeleton and is expressed by endothelial cells, smooth muscle cells, fibroblasts, and several immune cells [33].

The small proteins Myelin P2 protein and Acyl CoA binding proteins (ACBP) showed elevated levels in the OSP from intercostal muscle. Myelin P2 protein is involved in maintaining the structural integrity of myelin and plays a role in lipid metabolism within the nervous system [34]. ACBPs are involved in the transport and intracellular targeting of acyl-CoA molecules, which are important intermediates in lipid metabolism. They play a role in facilitating the movement of fatty acids within the cell for various cellular processes, including energy production [35]. They have been studied for their potential role in myelin stability and repair. Thus, Myelin P2 might have potential therapeutic applications in treating demyelinating diseases such as multiple sclerosis [36].

Metalloproteins like Metallothionein or copper chaperone antioxidant 1 were observed in liver and kidney OSPs. Metallothioneins are versatile proteins that are crucial in maintaining metal homeostasis within cells, protecting against heavy metal toxicity, and contributing to cellular defense mechanisms. Their functions extend to cellular processes such as proliferation, differentiation, and protection against oxidative stress [37]. They might have potential therapeutic applications in the treatment of metal toxicity and oxidative stress-related diseases [38].

Other small proteins highly expressed and related to oxidative stress are Glutaredoxin-1 and Thioredoxin. Glutaredoxin-1, also known as Grx1, is a small heat-stable protein that maintains the redox state of cells, which is vital for cellular function and survival. Grx1, specifically, helps reduce disulfides via the glutathione system, contributing to cellular redox homeostasis [39]. Its critical role in redox reactions suggests it may have a larger role in protecting cells from oxidative damage. 

Research has demonstrated that disruptions in redox homeostasis could contribute to the development of many diseases, including neurodegenerative disorders such as Alzheimer’s, Parkinson’s, and ALS. In these conditions, oxidative damage caused by reactive oxygen species is thought to play a significant role in nerve cell death. Given Grx1’s function in mitigating oxidative damage, it is being extensively studied for its potential therapeutic role in these diseases. 

Emerging studies also suggest that Grx1 may have other cellular functions beyond those related to redox regulation, such as modulating signal transduction pathways and potentially influencing processes like apoptosis and cell proliferation [40,41].

Thioredoxins are small proteins abundant in all OSPs and involved in redox signaling and regulation. They play a key role in maintaining cellular redox balance by reducing disulfide bonds in target proteins, contributing to cell survival and protection against oxidative damage. Thioredoxin is critical in maintaining the balance of redox reactions within cells. It is an antioxidant that reducing other proteins via cysteine thiol–disulfide exchange [42]. Thus, it is involved in maintaining the cell’s status and adapting to changes in the redox environment—an essential aspect of many biological processes, including DNA synthesis and repair and cell growth and death. It might have potential therapeutic applications in supporting immune responses under healthy conditions [43].

At the molecular level, thioredoxin aids in regulating the cellular redox environment by reducing oxidized cysteine residues on proteins, thereby regulating their activity. This has a broad impact, influencing the functions of multiple proteins, including transcription factors and other signaling molecules, affecting overall cellular processes such as differentiation, proliferation, and apoptosis.

Due to its roles in cell signaling and redox regulation, disruptions to thioredoxin function are implicated in various diseases. For instance, studies have found links to cancer, where elevated levels of thioredoxin have been observed. Its overexpression is thought to promote cellular proliferation and survival, potentially contributing to tumor growth. Moreover, it may also play a protective role in resistance to oxidative stress, which is associated with cancer progression.

Similarly, thioredoxin is also investigated for its role in neurodegenerative diseases, where maintaining redox balance is crucial for neuronal cell survival. Additionally, studies have suggested a potential role for thioredoxin in cardiovascular diseases, with its antioxidant properties possibly conferring protective effects. 

### 3.3. Proteins Found in OSP and Their Characterization

All tissues exhibited qualitatively similar protein profiles; however, significant quantitative differences were observed. Three distinct groups of OSPs were identified, displaying substantial variations between each other. Group 1 comprised the intercostal muscle, followed by Group 2, which included the kidney, and finally, group 3 encompassed all other analyzed tissues. Notably, while sharing similarities with group 3 (primarily involving fatty acid binding proteins and retinol binding protein 2), the kidney exhibited elevated thymosin expression rates typically found in thymus. 

The IM OSP displayed the most distinctive protein expression profile, primarily featuring proteins related to bone and muscle. Each tissue exhibited characteristic protein expression, except for the lungs OSP, where no protein demonstrated higher expression than other analyzed tissues. The heatmap for lung OSP revealed the downregulation of proteins typically upregulated in other tissues.

Tissue-enriched proteins were mostly found in kidney, OM, and IM OSPs. OM showed similar enrichment to the liver due to liver tissue being one of the parts in OM OSP. Kidney OSP showed a high expression rate and enrichment in a specific group of nuclear proteins, namely, the Heterogenous nuclear ribonucleoproteins. These are crucial in proper gene expression and RNA/DNA protection [44]. This specificity to the kidney could be related to the need to adapt to various stressors in which hnRNPs are highly involved. 

Glutathione S-Transferase Yc is a part of the family of enzymes known as glutathione S-transferases. These enzymes play a vital role in detoxifying foreign substances, notably reactive oxygen species and xenobiotics, by catalyzing the conjugation of the reduced form of glutathione to these compounds [45]. The process enhances the water solubility of these substances, thereby facilitating their removal from the body. By doing so, GST Yc participates in the metabolism of carcinogens, therapeutic drugs, environmental toxins, and products of oxidative stress, resulting in protection against cellular damage.

In addition to detoxification, GST Yc is thought to be involved in various other physiological roles, including regulating signal transduction pathways, tissue repair mechanisms, and even the progression of various diseases [45]. Some GSTs have been implicated in pathologies like cancer and neurodegenerative, cardiovascular, and respiratory diseases, as alterations in their activities can have profound effects on cell survival, proliferation, and death.

Through these important roles, Glutathione S-Transferase Yc contributes to maintaining cellular homeostasis and has become an area of focus in understanding the biology of stress reactions and detoxification pathways in the human body. For these reasons, GST Yc is of substantial interest in the context of both health and diseases. It has potential therapeutic applications in immune support and detoxification [45].

Another group of abundant proteins was proteins involved in the immune response. We already discussed Thymosins in the previous section; here, we focus mostly on bigger immunomodulating proteins. 

Prostaglandin E Synthase 3 is an enzyme involved in synthesizing prostaglandin E2, a lipid compound that plays a significant role in mediating inflammation and pain responses in the body. PTGES3 catalyzes the conversion of prostaglandin H2 to PGE2, the latter of which is a principal mediator of inflammation and can induce fever, swelling, and pain [46,47].

PGE2 has a wide range of biological effects, including vasodilation, increased vascular permeability, and the sensitization of nerve endings to pain. It helps to regulate the immune response and assists in the repair and healing of tissues after injury. However, elevated levels of PGE2 have been found in various pathological conditions, including inflammatory diseases, cancer, and neurodegenerative disorders such as Alzheimer’s disease.

Given its critical role in various pathophysiological conditions, the precise regulation of PGE2 synthesis by PTGES3 makes this enzyme a potential therapeutic target. Modulating the activity of PTGES3 may alleviate symptoms associated with high PGE2 levels, such as inflammation and pain, presenting significant opportunities for developing novel therapeutic approaches to treat these conditions.

Hence, PTGES3 continues to gain attention in biomedical research, especially in inflammation, pain modulation, and the search for therapeutic targets for various diseases.

Peptidyl-Prolyl cis-trans Isomerase FKBP3 is an enzyme that assists in the proper folding of proteins. This function is critical to ensuring that proteins achieve their necessary three-dimensional structures, which are essential for their function. The correct folding of proteins is vital for various biological processes, including cellular growth, differentiation, and response to environmental stimuli [48].

One of the unique aspects of FKBP3 is its contribution to immune response modulation. The FKBP family of proteins are known to possess immunosuppressive properties. They achieve this by binding to immunosuppressive drugs like tacrolimus and sirolimus (rapamycin). The complexes formed by FKBP–drug binding can inhibit the activity of certain proteins pivotal for immune response, such as the protein kinase mTOR (mammalian Target Of Rapamycin). By inhibiting mTOR, these complexes can suppress the immune response, which can be beneficial in preventing organ transplant rejection and treating autoimmune diseases.

FKBP3’s dual roles in protein folding and immune response modulation make it critical to various cellular processes. Therefore, there has been an increasing interest in studying this protein, especially in pathologies where protein folding and immune regulation are disrupted. It has potential therapeutic applications in treating muscular disorders [46].

Beta-2-Microglobulin (B2M) is an integral component of Major Histocompatibility Complex Class I molecules, which are crucial for immune response. MHC Class I molecules are found on the surfaces of all nucleated cells and play a fundamental role in the immune system’s ability to recognize and destroy infected or malignant cells [49].

The MHC class I molecule is a heterodimer consisting of heavy and light chains (B2M). In this complex, B2M stabilizes the structure and aids in presenting peptide antigens to the immune system. These antigens, displayed on the cell surface, are recognized by T-cells—a type of white blood cell critical for immune responses. Suppose the T-cells recognize the antigens as foreign (as in viral or bacterial infections) or abnormal (as in cancer). In that case, they trigger an immune response, destroying the affected cell.

Besides its role in immune surveillance, B2M has other implications. Elevated levels of B2M in the bloodstream can indicate certain conditions like chronic inflammation, some types of cancer, or kidney dysfunction. In the field of transplantation, B2M mismatches can lead to transplant rejection. Moreover, B2M is involved in a condition called dialysis-related amyloidosis, where it forms amyloid fibrils that can lead to complications in patients undergoing long-term dialysis.

Given its role in immunity and potential as a disease marker, Beta-2-Microglobulin continues to be an important area of research in understanding the immune system and various disease processes. It has potential therapeutic applications in treating leukemia, other types of cancer, and immune diseases [49]. 

Another important protein found in OSPs in large abundance and in all tissues is Carbonic Anhydrase 2. It plays a crucial role in pH regulation and carbon dioxide transport. There are also some studies suggesting its role in bone resorption. It has potential therapeutic applications in the treatment of glaucoma [50].

### 3.4. Outlook for the Proteomic Characterization of OSPs

Using proteomic approaches to identify and characterize OSPs is a valuable technique in understanding the protein composition and potential biological functions associated with different organs. We used a general bottom-up proteomics approach to obtain functional insights into OSPs. Up to now, there has been a lack of up-to-date literature that characterizes the proteome, including small proteins and protein fragments obtained from organs and tissues of rabbit pups using ultrafiltration methods. Our study employed soft homogenization, ultrafiltration, and size exclusion via ultrafiltration for protein isolation. Soft homogenization is used to break down tissues gently, allowing for the release of proteins while minimizing degradation. Size exclusion ultrafiltration is a technique that separates proteins based on their molecular weight by passing the sample through a membrane with a specific molecular weight cutoff. In this study, we chose to use a benchtop system with a peristaltic pump so that multiple filters with different MWCO can be used after each other so as not to block up a lower MWCO cassette with big proteins and thus not allow smaller proteins to pass through. Gel electrophoresis and mass spectrometry were utilized for proteome analysis. Gel electrophoresis separates proteins based on size and charge, allowing for the visualization and preliminary characterization of protein bands. On the other hand, mass spectrometry is a powerful technique for identifying and quantifying proteins by measuring their mass-to-charge ratios. Gene ontology analysis was applied to the mass spectrometry data to categorize proteins based on their functions and biological processes. This analysis helps us gain insights into the roles and interactions of the identified proteins within specific cellular pathways or processes.

It is important to note that the specific methods used in this study may vary from those in other studies, and the effectiveness of the techniques may depend on various factors such as sample preparation, instrument sensitivity, and data analysis algorithms [51,52,53,54,55,56,57,58,59,60,61]. 

By examining the methods employed in these studies, we can observe the diversity of techniques used in proteomic research. Mass spectrometry is a prevalent method used across multiple studies, as it identifies and quantifies proteins and peptides. It offers high sensitivity and specificity, making it a valuable tool in proteomics. Overall, in comparing our study with others, these studies showcase the wide range of techniques employed in proteomic research, highlighting the importance of selecting appropriate methods based on the research objectives and the characteristics of the samples being studied. Advanced technologies, such as mass spectrometry, have greatly expanded our ability to analyze complex protein mixtures and elucidate their functional roles in various physiological and pathological processes.

Our approach used a standard workflow for protein identification, FDR estimation, and quantification, which might be less useful for small protein discovery, including efficient and reliable detection and analysis. To discover more new small proteins in OSPs, more targeted proteomics approaches could be considered in future research.

## 4. Materials and Methods

### 4.1. Production of Organ-Specific Protein Ultrafiltrates (OSP)

Postmortem frozen laboratory-grade rabbit pups (no more than −20 °C) were received, thawed, washed, and dried off. The pups obtained were laboratory-grade broiler rabbits bred in the Institute of Small Farm Animals at the Research Institute of Animal Production Nitra, Slovakia. The population of M91 broiler rabbits is being maintained in broiler rabbit production systems by interline hybridization from the NZW (New Zealand White) rabbit strain. The rabbit pups were put down in the institute’s facilities by Slovakian law based on the Act of 12 December 2006 on veterinary care 39/2007 Coll., after a medical examination and under veterinary supervision by professionally qualified personnel. 

The organs were taken out, combined with saline solution in sterile test tubes, homogenized, and centrifuged in a cooled centrifuge for 10 min at 6000 rpm, followed by pre-filtration through ceramic filters. After pre-filtration, the samples were flash-frozen to −70 °C for 24 h. Using a tabletop crossflow system with a cassette filter, the samples were then microfiltered with a cassette with a MWCO 300 kDa and 100 kDa and then ultrafiltered with a cassette with a MWCO 50 kDa. The final permeate was gathered into sterile medium flasks for usage. The production process was standardized and carried out on ice in a cold environment. At last, the concentrations of all OSPs were measured with a standard BCA assay protein kit following the manufacturer’s instructions. This process was repeated for each organ at least 3 times to ensure the higher validity of the results (see Table 2). 

### 4.2. PAGE Gels According to Schägger

The sample (5 µg up to a maximum of 12 µL) was mixed with 4× Laemmli sample buffer (250 mM tris, pH 6.8; 12% glycerol; 4% SDS; 10% beta-mercaptoethanol; 0.05% bromphenol blue), then boiled for five minutes at 95 °C. By [62], samples were separated on a gel after cooling (7 × 8 cm; 4% stacking gel consisting of 4% acrylamide:bisacrylamide (29:1); 68 mM tris, pH 6.8; 0.2% SDS; 0.2% TMED; 0.03% APS and 18% separating gel consisting of 18% acrylamide:bisacrylamide (32:1); 1M tris, pH 8.45; 0.1% SDS; 14% glycerine; 0.05% TMED; 0.05% APS) at 150 Volt for 180 min (Mini-protein II Dual Slab Cell, Bio-Rad, Shinagawa, Tokyo). The protein sizes were calculated by comparing the migration of the protein band to a molecular mass standard (Mark12, Thermo, Waltham, MA, USA) after the gel was stained with MS-compatible silver staining (Proteome Factory PS-2001, Berlin, Germany). The gel was scanned using a PowerLook 2100 XL scanner (Dallas, TX, USA) with a transparency adaptor at a resolution of 150 dpi.

### 4.3. Liquid Proteolysis

Liquid proteolysis was typically performed by solubilization of an amount of 6 µL protein into 10 µL 8 M urea. Subsequently, 5 mM (final) of TCEP was added for reduction, which was performed for 20 min at room temperature before alkylation by the addition of 10 mM (final) iodoacetamide (IAA). The latter was performed for 20 min at room temperature in the dark. After alkylation, the sample was diluted 1:10 by adding 50 mM TEAB buffer. Then, 100 ng trypsin was added, and the proteolysis was incubated at 37 °C overnight. After the overnight incubation, a second addition of another 100 ng trypsin was made. The additional proteolysis step was incubated for another 2 h (RT) before the reaction was stopped by adding 0.5% (final) formic acid before MS analysis.

### 4.4. Liquid Chromatography Mass Spectrometry/Mass Spectrometry (LC-MS/MS) Analysis

The LC-MS/MS analysis of OSP protein/peptides was performed using an Ultimate 3000 nano HPLC system for chromatographic separation coupled via a nanoelectrospray interface to an Orbitrap Velos. Water served as solvent A, and acetonitrile served as solvent B (with 0.1% formic acid additions). Aliquots of 200 ng proteolytic peptides were loaded onto a trapping column (kept at 35 °C) for desalting (Thermo, Dionex, Sunnyvale, CA, USA, Pepmap C18) before peptides were separated by gradient elution on a 500 × 0.075 mm column (0.5 µL/min, held at 50 °C, Reprosil C18-AQ, Dr. Maisch, Ammerbuch, Germany) from 12% to 40% B. The column effluent was directed to an Orbitrap Velos mass spectrometer via a nanoelectrospray ion source. Up to 10 MS/MS spectra from ions of interest (charge states +2 and higher) were data-dependently captured in the instrument’s linear ion trap. At the same time, survey scans were detected at a notional resolution of R = 60,000. Each analysis’s total acquisition time was 150 min. 

### 4.5. Database Search with MaxQuant

The protein identification was performed with MaxQuant (https://www.maxquant.org/, accessed on 21 August 2023) using an FDR of 1%. Proteome Factory AG conducted the protein identification, and the MaxQuant results were provided to MicroDiscovery GmbH. We removed all contaminants and hits from the reverse database. We selected all proteins with at least two proteins. For protein quantification, we used the LFQ intensities.

### 4.6. Bioinformatics

The data were generated in four experiments: 9J, 10J, 11J, and 12J (Table 2). A preliminary experiment 7J was prepared to choose the optimal setup for the main runs. The datasets contain proteins measured in 6 different tissues/tissue mixtures (Table 2).

Gene Ontology (GO) is used to identify functional components using all three branches of the GO tree. We utilized the R packages “biomaRt” [63] using Ensembl with dataset “*ocuniculus* gene ensembl” to map proteins to GO terms. We additionally collected GO annotation using the UniProt web service R package (version 2.22.0) to analyze the pathway based on mapping the proteins to the corresponding genes, as the number of proteins with GO annotation was limited using biomaRt. Functional components were found using an over-representation approach while utilizing the KEGG pathways repository, and we also examined other pathways. We employed “biomaRt”, “KEGG.db”, and “KEGGREST” from R packages for mapping proteins and pathways to identify enriched pathways [64] via over-representation analysis.

To analyze organ-related GO terms, including Molecular Process, Cellular Component, and Molecular Function, the respective samples from the different tissues/organs were treated independently. Furthermore, the tissue-specific analyses of proteins found exclusively in a tissue were discriminated. In addition to the analyses based on protein IDs, we also investigated the quantification data (LQF intensities). Here, we only considered proteins measured in at least 50% of the respective samples and 33% of the others. MA plots with quantification values after the logarithmic transformation of all OSPs were generated. In addition, heat maps of the top 50 proteins with the highest differences between the tissue and the rest were provided.

The mass spectrometry proteomics data have been deposited into the ProteomeXchange Consortium via the PRIDE [65] partner repository with the dataset identifiers PXD050039 and 10.6019/PXD050039.

### 4.7. Finding the Tissue-Specific Proteins/Genes with Uniprot, g: Profiler and Human Protein Atlas

For more insight into organ-specific genes and SPs obtained in samples, we used the web-based software UniProt and g: Profiler (https://biit.cs.ut.ee/gprofiler/gost, accessed on 21 August 2023). The genes were mapped from the mass spectrometry data using UniProt. They were subsequently converted to human genes in the g: Profiler engine and categorized according to their tissue affinity using a protein database from HPA as a data source. The organ-specific genes/proteins were extracted and separated according to their HPA term.

### 4.8. Study Limitations

There are inherent limitations involved with working with natural biological material. With every extraction, even when standardized, there will not be a completely exact sample produced. This is due to the dynamics of protein expression within each living organism. This can vary across tissues within one organism and between two individuals of the same species. One can, however, try to come as close as possible. As one of the measures to make the sample preparation as close as possible for each sample, we took measures to keep the temperatures as low as possible to ensure the optimal working conditions of the samples. 

Because one of the goals was to determine naturally occurring proteins, no additional inhibitors were added to the samples during or after preparation. This can also explain some variations between different runs due to different protein degradation rates.

## 5. Conclusions

The findings of this study have uncovered the therapeutic potential of proteins/SPs that could contribute to various interventions. Identifying a diverse range of proteins that play crucial roles in relevant pathways highlights the significance of SPs in this context. These SPs might potentially enhance tissue repair and act as key factors in providing biomolecules for tissue physiology, repair, and rejuvenation. Considering the projected growth of the aging population, utilizing SPs as an alternative strategy holds promise for improving overall health and preserving quality of life in the future. Also, showing organ-specific activity in OSPs could unlock possible therapeutic applications.

In this study, the samples were characterized using proteomic techniques. However, it is important to note that the results obtained from mass spectrometry were semi-quantitative, and further validation is required. Moreover, the presence of blood-specific proteins could not be avoided, which might have resulted in a reduced number of proteins detected by the mass spectrophotometer. It is worth mentioning that the study was designed to map naturally occurring proteins, including small proteins, and therefore, no protease-inhibiting substances were added. Although low temperatures were maintained throughout the experiment to inhibit the activity of endogenous proteases, naturally occurring proteolysis likely occurred during the homogenization and filtration processes. Before ultrafiltration, the bigger proteins found in the samples could thus be fragmented, pass through smaller MWCO, and then be detected by mass spectrometry. Lastly, mass spectrometry in this study has contributed to the overall analysis of the obtained results. 

It has to be mentioned that we did not determine the exact extent of proteolysis caused by endogenous proteases. The information obtained from protein gels was the primary source used to assess the level of proteolysis. However, to validate the presence of specific unique SPs/proteins and confirm the potential effects of the ultrafiltrates, both in vitro and in vivo validation tests should be conducted. These tests will provide additional evidence and further support the speculations regarding the effects of the ultrafiltrates. 

This study shows that fetal tissues and organs are highly complex and contain various cell types with networks of bioactive molecules. SPs/proteins from such tissues require extensive characterization and quality control to ensure the safety and efficacy of the resulting therapeutic products.

In summary, the proteomic analysis of tissue extracts is a powerful tool for discovering proteins, peptides, and protein fragments that may have clinical relevance. By investigating the protein composition of organs/tissues in developmental and adult stages, we can uncover molecules and networks of biomolecules that can be further studied and potentially translated into pre-clinical and clinical applications, leading to improved individualized treatment.

## Figures and Tables

**Table 1:** Proteins found exclusively in listed OSPs (SPs are underlined).

OSP	Proteins
Liver	U2A’/phosphoprotein 32 family A C-terminal domain-containing protein
Lung	Cytochrome P450, family 4, subfamily A, polypeptide 5 LLGL scribble cell polarity complex component 1 Pleckstrin homology and RhoGEF domain containing G4B Coiled-coil domain-containing protein
Intercostal muscle	Protein S100-A11 (Calgizzarin) Thioredoxin 2 Myozenin 2 Thioredoxin domain-containing protein Phosphoglucomutase-1 Zinc finger MYM-type containing 2 Collagen type XI alpha 1 chain
Kidney	Peptidyl-prolyl cis-trans isomerase E (PPIase E) Septin Stress induced phosphoprotein 1 General transcription factor IIi Centromere protein E
Organ mix	Small muscular protein Galectin Plectin/eS10 N-terminal domain-containing protein Crystallin gamma N Beta-crystallin A2 (Beta-A2 crystallin) Crystallin beta B3 Crystallin beta A1 Crystallin beta B1 Histidine-rich glycoprotein (HPRG) DCC netrin 1 receptor Spectrin repeat containing nuclear envelope protein 1

**Table 2:** OSP ultrafiltrate samples from different tissues/organs and runs were used in this study.

Sample	7J ^1^	9J	10J	11J (1)	11J (2)
Liver	LI 7J-MFP1	LI 9J-50-22	LI 10J-50-4	LI 11J-50-20	LI 11J-50-23
	LI 7J-MFP2				
	LI 7J-UF1P1				
	LI 7J-UF1P2				
	LI 7J-UF2P1				
	LI 7J-UF2P2				
Lung		LU 9J-50-22	LU 10J-50-4		LU 11J-50-23
Intercostal muscle		IM 9J-50-22	IM 10J-50-4	IM 11J-50-20	
Kidney		KI 9J-50-22	KI 10J-50-4	KI 11J-50-20	
Organ Mixture ^2^		OM 9J-50-22	OM 10J-50-4	OM 11J-50-20	OM 11J-50-23

^1^ Preliminary runs for choosing better cut-offs for ultrafiltration. ^2^ Organ mixture = liver, pancreas, placenta, stomach, intestinal mucosa, kidney, and eye.

## Supplementary Materials

The supporting information can be downloaded at https://www.mdpi.com/article/10.3390/ijms25052863/s1.
