# Supplementary material for: Exploring the Molecular Tapestry: Organ-Specific Peptide and Protein Ultrafiltrates and Their Role in Therapeutics
Source: Int J Mol Sci. 2024 Mar 1;25(5):2863. doi: 10.3390/ijms25052863 (PMC10931619; doi:10.3390/ijms25052863)
Supplement: Supplementary file 1 [file ijms-25-02863-s001.zip › ijms-2838146-supplementary.pdf]

Supplementary Table S1. List of mapped tissue-specific genes (from all samples joint for each organ).

OSPU	Genes
**Liver**	COX17, MIF, FAM107B, SRSF3, DSTN, HNRNPAB, PEBP1, PARK7, RAB5A, H1-3, LXN, FKBP3, SNRPB2, YWHAG, EMD, CA2, LYPLA1, PABPN1, NUCKS1, YWHAE, HNRNPC, TST, CALU, MDH2, APEX1, CTSB, RALY, SLC9A3R1, PCBP1, NSFL1C, RAD23B, HNRNPDL, PAICS, HNRNPH1, NXF1, TUBA1C, HNRNPK, PACSIN2, KRT8, PSAP, FUS, PDZK1, ATP5F1B, PSIP1, CTTN, SFPQ, BIN1, HNRNPU, HSPD1, EZR, KARS1, LMNB1, DYNC1I2, LMNA, NCL, HSP90AB1, CPSF6, HNRNPL, SF3A1, SF3B2, ILF3, THRAP3, BCLAF1, DDX46, RRBP1, SPTAN1, FLNB, DBI, H1-4, RGN, PCBP2, ACAA2, HMGCS2, SCP2, LBR, CPS1, UFM1, PFN1, TXN, B2M, TAX1BP3, BLVRB, CCDC12, MTPN, CFL2, SH3BGRL, RAB1A, RAB7A, RAB14, GRB2, SRSF9, CWC15, COMT, EEF1D, HNRNPD, TXNL1, CFDP1, AIMP1, LASP1, ELAVL1, SERBP1, TUFM, EEF1A1, VIM, PDIA4, ADAR, UBAP2L
**Lungs**	APEX1, BIN1, PARK7, B2M, RAB7A, GAPDH, VIM, NCL, HNRNPK, H1-4, CALU, VPS13A, ATP5F1B, LMNA, EPRS1, HSPD1, NUCKS1, FUS, SF1, HNRNPC, HNRNPD, SFPQ, TUFM, TARDBP, FUBP3, SRSF3, LMNB1, SERBP1, PSIP1, CCAR1, ILF3, NXF1, HNRNPL, PSAP, LASP1, PDCD5, EMD, FLNB, BLVRB, SF3A1, SF3B2, DYNC1I2, CTTN, HNRNPDL, HNRNPU, RALY, RRBP1, RAD23B, U2SURP, TST, AK1, EZR, DDX17, DDX46, HCLS1, NPC2, SNRPB2, SPTAN1, MDH2, STBD1, CELF2, THRAP3, ADAR, POSTN, SCP2, CYB5A, COMT, FN1, PFN1, AK2, FABP5, DBI, ARHGDIB, AIMP1, TXN, LLGL1, HCFC1, CBX5, FKBP3, SRSF9, NCK1, LYZ, LCP1, CST3, LBR, CTSB, CEP83, TUBA1C, YWHAG, UFM1, HNRNPAB
**Intercostal muscle**	COX17, MB, SRSF3, HNRNPAB, CSRP3, MYL1, HSPB6, H1-4, H1-3, CFL2, AK1, SARNP, MYOZ2, HNRNPD, MYOZ1, HNRNPC, NUCKS1, TARDBP, CA3, PDLIM3, STBD1, ACTA1, HNRNPH1, NXF1, EEF1A1, HNRNPK, FUS, BIN1, SFPQ, HNRNPU, SF1, LBR, PDLIM5, NCL, CPSF6, LMNA, HNRNPL, SF3A1, MYOT, ILF3, THRAP3, MYBPC1, CCAR1, FLNC, NEB, SNCA, B2M, LYZ, BLVRB, TXN2, TPT1, CAMP, MTPN, ARHGDIB, YWHAE, SERBP1, PAICS, TUBA1C, VIM, PSAP, CELF2, KARS1, LMNB1, LCP1, SF3B2, RRBP1
**Kidney**	SLC9A3R1, B2M, VIM, NCL, HNRNPK, H1-4, FN1, ZEB1, LMNA, HSPD1, CPSF6, NUCKS1, FUS, SF1, HNRNPC, HNRNPD, SFPQ, TARDBP, SRSF3, RAD23B, LMNB1, SARNP, SPTAN1, CCAR1, ILF3, BCLAF1, NXF1, HNRNPL, PSAP, H1-3, EHD1, LASP1, PQBP1, FLNB, GTF2I, HNRNPDL, HNRNPU, HNRNPH1, DSTN, HNRNPAB, RALY, NCK1, CYB5A, GAPDH, CA2, PDZK1, RGN, ASRGL1, SATB2, ALB, PEBP1, CENPE, FKBP2, NPC2, SNRPB2, TUFM, PCK2, MAPT, MDH2, FABP3, CTSB, STBD1, AKR1B10, POSTN, HDLBP, TST, BIN1, PARK7, RAB7A, EZR, CALU, ATP5F1B, RAB5A, DDX46, UTRN, ELAVL1, MIF, TPT1, EEF1D, LBR, KYAT3, CWC15, GMFB, ARHGDIB, FUBP3, CFDP1, PABPN1, SERBP1, PSIP1, NSFL1C, CEP290, CEP83, SH3BGRL3, YWHAG, AIMP1, EMD, YWHAE, THRAP3, SF3A1, PDLIM5, SF3B2, DYNC1I2, CTTN, CCDC12, CBX5, SH3BGRL, COL15A1, UBAP2L, RRBP1, EFHD2, HMGCS2, CELF2, STX7, STIP1, H1-1
**Organ Mixture**	APEX1, B2M, SCP2, VIL1, NCL, CA2, PDZK1, HMGCS2, LMNA, HSPD1, FUS, EEF1D, PEBP1, TUFM, PCK2, AGR2, KRT8, PSIP1, ILF3, STBD1, AKR1B10, LASP1, FABP7, RBP2, LGALS2, FABP2, CELF2, HDLBP, RRBP1, PARK7, HSP90AB1, SPINK4, LYZ, VIM, LCP1, ARHGDIB, NPC2, LDB3, ATP5F1B, HNRNPC, SFPQ, SRSF3, CCAR1, HNRNPL, PSAP, PQBP1, FLNB, YWHAE, THRAP3, SF3A1, TST, HNRNPAB, SLC9A3R1, BIN1, EZR, HNRNPK, FKBP3, RAB5A, DDX46, PAICS, CPSF6, MIF, PCBP1, SNRPB2, RAD23B, LYPLA1, LMNB1, PABPN1, SPTAN1, NSFL1C, MDH2, PACSIN2, BCLAF1, CTSB, TUBA1C, NXF1, YWHAG, COX17, H1-3, EMD, SF3B2, DYNC1I2, CTTN, HNRNPDL, HNRNPU, HNRNPH1, DSTN, RALY, H1-4, RGN, SF1, DBI, LBR, ACAA2, PCBP2, RAB7A, EEF1A1, COMT, PFN1, PDIA4, RAB14, ELAVL1, RAB1A, TAX1BP3, TXN, SYNE1, HNRNPD, CWC15, GRB2, SERBP1, CFL2, SARNP, AIMP1, UFM1, BLVRB, MTPN, ADAR, SH3BGRL, UBAP2L, TXNL1, SRSF9, FN1, SNCA, EHD1, GAPDH, GMFB, FUBP3, CEP290, SH3BGRL3, PDLIM5, CBX5, COL15A1, EFHD2, NCK1, CYB5A, HRG, ASRGL1, ALB, FKBP2, PLIN2, FABP3, POSTN, STX7, H1-1, CRYBA2, CRYGD, CRYBB3, CRYGA, CRYBA1, CRYBB1, AK2, GLRX, ACTB, AAMDC

Supplementary Table S2. Overview of the tissue specific GO:terms analysis.

**OSPU**	**GO:terms**
Liver	GO:0030168: platelet activation<br>GO:0065008: regulation of biological quality<br>GO:0050878: regulation of body fluid levels<br>GO:0051489: regulation of filopodium assembly<br>GO:1901362: organic cyclic compound biosynthetic process<br>GO:0005811: lipid droplet<br>GO:0006996: organelle organization
Lungs	GO:0035613: RNA stem-loop binding<br>GO:0090571: RNA polymerase II transcription repressor complex<br>GO:0031333: negative regulation of protein-containing complex assembly<br>GO:0099572: postsynaptic specialization<br>GO:0016071: mRNA metabolic process<br>GO:0010941: regulation of cell death<br>GO:0018108: peptidyl-tyrosine phosphorylation<br>GO:0006952: defense response<br>GO:0018212: peptidyl-tyrosine modification<br>GO:0019058: viral life cycle<br>GO:0002694: regulation of leukocyte activation<br>GO:0007275: multicellular organism development<br>GO:0045087: innate immune response<br>GO:1903706: regulation of hemopoiesis<br>GO:0009636: response to toxic substance<br>GO:0061041: regulation of wound healing<br>GO:0002262: myeloid cell homeostasis<br>GO:0043254: regulation of protein-containing complex assembly<br>GO:0002253: activation of immune response<br>GO:0019901: protein kinase binding<br>GO:1903034: regulation of response to wounding<br>GO:1902236: negative regulation of endoplasmic reticulum stress-induced intrinsic apoptotic signaling pathway<br>GO:0019722: calcium-mediated signaling<br>GO:0044089: positive regulation of cellular component biogenesis<br>GO:0045602: negative regulation of endothelial cell differentiation
Intercostal Muscle	GO:0072673: lamellipodium morphogenesis<br>GO:0006879: cellular iron ion homeostasis<br>GO:0032946: positive regulation of mononuclear cell proliferation<br>GO:0010039: response to iron ion<br>GO:0035304: regulation of protein dephosphorylation<br>GO:0055072: iron ion homeostasis<br>GO:0020037: heme binding<br>GO:0046906: tetrapyrrole binding<br>GO:0070665: positive regulation of leukocyte proliferation<br>GO:0043535: regulation of blood vessel endothelial cell migration<br>GO:0016050: vesicle organization<br>GO:0048306: calcium-dependent protein binding<br>GO:0006950: response to stress<br>GO:0098771: inorganic ion homeostasis<br>GO:0043068: positive regulation of programmed cell death

	GO:0046651: lymphocyte proliferation<br>GO:0032943: mononuclear cell proliferation<br>GO:0050801: ion homeostasis<br>GO:0016236: macroautophagy<br>GO:0030060: L-malate dehydrogenase activity<br>GO:0006869: lipid transport<br>GO:0071705: nitrogen compound transport<br>GO:0002367: cytokine production involved in immune response<br>GO:0002718: regulation of cytokine production involved in immune response<br>GO:0006091: generation of precursor metabolites and energy<br>GO:2000269: regulation of fibroblast apoptotic process<br>GO:0016853: isomerase activity<br>GO:0003906: DNA-(apurinic or apyrimidinic site) endonuclease activity<br>GO:0007155: cell adhesion<br>GO:0005496: steroid binding<br>GO:0016868: intramolecular transferase activity, phosphotransferases<br>GO:0070885: negative regulation of calcineurin-NFAT signaling cascade<br>GO:0106057: negative regulation of calcineurin-mediated signaling
Kidney	GO:0048646: anatomical structure formation involved in morphogenesis<br>GO:0009790: embryo development<br>GO:0048524: positive regulation of viral process<br>GO:0006886: intracellular protein transport<br>GO:0044085: cellular component biogenesis<br>GO:0001525: angiogenesis
Organ Mixture	GO:0043537: negative regulation of blood vessel endothelial cell migration<br>GO:0046983: protein dimerization activity<br>GO:0016408: C-acyltransferase activity<br>GO:0004033: aldo-keto reductase (NADP) activity<br>GO:0043891: glyceraldehyde-3-phosphate dehydrogenase (NAD(P)+) (phosphorylating) activity<br>GO:0004365: glyceraldehyde-3-phosphate dehydrogenase (NAD+) (phosphorylating) activity<br>GO:0031639: plasminogen activation<br>GO:0005996: monosaccharide metabolic process<br>GO:0050922: negative regulation of chemotaxis<br>GO:0006935: chemotaxis<br>GO:0120032: regulation of plasma membrane bounded cell projection assembly<br>GO:0060491: regulation of cell projection assembly<br>GO:0007167: enzyme-linked receptor protein signaling pathway<br>GO:0010638: positive regulation of organelle organization<br>GO:0007169: transmembrane receptor protein tyrosine kinase signaling pathway

Supplementary Table S3. List of all small proteins found.

10 kDa heat shock protein, mitochondrial (10 kDa chaperonin) (Chaperonin 10)
14 kDa phosphohistidine phosphatase (EC 3.9.1.3) (Phosphohistidine phosphatase 1) (PHPT1) (Protein histidine phosphatase) (PHP)
Acyl-CoA-binding protein (ACBP) (Diazepam-binding inhibitor) (DBI) (Endozepine) (EP)
Acylphosphatase-2 (EC 3.6.1.7) (Acylphosphatase, muscle type isozyme) (Acylphosphate phosphohydrolase 2)
Adipogenesis associated Mth938 domain containing
Alpha-lactalbumin (Lactose synthase B protein)
Alpha-synuclein
Antioxidant 1 copper chaperone
ATP synthase-coupling factor 6, mitochondrial (ATPase subunit F6)
Beta-2-microglobulin
BolA family member 2
Calmodulin (CaM)
Caveolae associated protein 1
Cellular retinoic acid binding protein 1
Costars family protein ABRACL (ABRA C-terminal-like protein)
Cystatin-C (Cystatin-3)
Cytochrome b5
Cytochrome c domain-containing protein
Cytochrome c oxidase copper chaperone COX17
Dynein light chain 1, cytoplasmic (8 kDa dynein light chain) (DLC8) (Dynein light chain LC8-type 1) (Protein inhibitor of neuronal nitric oxide synthase) (PIN)
EF-hand domain family member D2
Enhancer of rudimentary homolog
Eukaryotic translation initiation factor 4C
Eukaryotic translation initiation factor 5A (eIF-5A)
Family with sequence similarity 107 member B
Fatty acid binding protein 2
Fatty acid binding protein 3
Fatty acid binding protein 5
Fatty acid-binding protein, liver (L-FABP) (Liver-type fatty acid-binding protein)
Galectin
Glia maturation factor
Glutaredoxin-1 (Thioltransferase-1) (TTase-1)
GTP-binding nuclear protein Ran
Hemoglobin subunit alpha-1/2 (Alpha-1/2-globin) (Hemoglobin alpha-1/2 chain)
Lysozyme
Lysozyme (Lysozyme F1)
Lysozyme C (EC 3.2.1.17) (1,4-beta-N-acetylmuramidase C)
Macrophage migration inhibitory factor
Mammalian defensins domain-containing protein
Metallothionein-2E (MT-2E) (Metallothionein-IIE) (MT-IIE)

Myelin P2 protein
Myoglobin
NEDD8 (Neddylin) (Ubiquitin-like protein Nedd8)
Peptidyl-prolyl cis-trans isomerase (PPIase) (EC 5.2.1.8)
Peptidyl-prolyl cis-trans isomerase FKBP1A (PPIase FKBP1A) (EC 5.2.1.8) (12 kDa FK506-binding protein) (12 kDa FKBP) (FKBP-12) (Calstabin-1) (FK506-binding protein 1A) (FKBP-1A) (Immunophilin FKBP12) (Rotamase)
peptidylprolyl isomerase (EC 5.2.1.8)
Profilin
Protein MIX23 (Coiled-coil domain-containing protein 58)
Protein S100-A11 (Calgizzarin) (Protein S100-C) (S100 calcium-binding protein A11)
Prothymosin alpha
Retinol binding protein 2
Serine peptidase inhibitor, Kazal type 4
SH2 domain-containing protein 1A
SH3 domain-binding glutamic acid-rich-like protein
Small acidic protein
Small muscular protein
Small ubiquitin-related modifier (SUMO)
Sorting nexin 3
Stathmin
Tax1-binding protein 3
Thioredoxin (Trx)
Thioredoxin domain-containing protein 17
Thymosin beta
Thymosin beta-4 (T beta 4) [Cleaved into: Hematopoietic system regulatory peptide (Seraspenide)]
Tubulin-specific chaperone A
UBC core domain-containing protein
Ubiquitin-fold modifier 1
WH1 domain-containing protein
Whey acidic protein (WAP)
